# Influence of oral processing behaviour and bolus properties of brown rice and chickpeas on in vitro starch digestion and postprandial glycaemic response

**DOI:** 10.1007/s00394-022-02935-7

**Published:** 2022-06-30

**Authors:** Yao Chen, Markus Stieger, Edoardo Capuano, Ciarán G. Forde, Sandra van der Haar, Meeke Ummels, Heleen van den Bosch, Rene de Wijk

**Affiliations:** 1 Food Quality and Design, Wageningen University & Research, Wageningen, The Netherlands; 2 Division of Human Nutrition and Health, Sensory Science and Eating Behaviour, Wageningen University & Research, Wageningen, The Netherlands; 3 Food & Biobased Research, Fresh Food Chains, Food, Health & Consumer Research, Wageningen University & Research, Wageningen, The Netherlands

**Keywords:** Chewing time, In vitro starch digestion, Postprandial glycaemic response, Brown rice, Chickpeas

## Abstract

**Purpose:**

Oral processing behaviour may contribute to individual differences in glycaemic response to foods, especially in plant tissue where chewing behaviour can modulate release of starch from the cellular matrix. The aim of this study was to assess the impact of chewing time of two starch based foods (brown rice and chickpeas) on bolus properties, in vitro starch digestion and postprandial glycaemic excursion in healthy subjects.

**Methods:**

In a cross-over trial participants (*n* = 26) consumed two carbohydrates-identical test meals (brown rice: 233 g; chickpeas: 323 g) with either long (brown rice: 41 s/bite; chickpeas: 37 s/bite) or short (brown rice: 23 s/bite; chickpeas: 20 s/bite) chewing time in duplicate while glycaemic responses were monitored using a continuous glucose monitoring device. Expectorated boli were collected, then bolus properties (number, mean area, saliva amylase activity) and in vitro starch digestion were determined.

**Results:**

Longer chewing resulted in significantly (*p* < 0.05) more and smaller bolus particles, higher bolus saliva uptake and higher in vitro degree of intestinal starch hydrolysis (DH_S_chewing time_%) than shorter chewing for both foods (brown rice: DH_S%_23 s_ = 84 ± 4% and DH_%S_41s_ = 90 ± 6%; chickpeas: DH_S%_20 s_ = 27 ± 3% and DH_%S_37s_ = 34 ± 5%, *p* < 0.001). No significant effect of chewing time on glycaemic response (iAUC) (*p* > 0.05) was found for both meals. Brown rice showed significantly and considerably higher in vitro degree of intestinal starch hydrolysis and glycaemic response (iAUC) than chickpeas regardless of chewing time. No significant correlations were observed between bolus properties and in vitro starch hydrolysis or glycaemic response (*p* > 0.05).

**Conclusion:**

Differences in the innate structure of starch based foods (brown rice compared to chickpeas) have a larger effect on postprandial glucose response than differences in mastication behaviour although oral processing behaviour showed consistent effects on bolus properties and in vitro starch digestion.

**Trial registration** ClinicalTrials.gov identifier: NCT04648397 (First posted: December 1, 2020).

**Supplementary Information:**

The online version contains supplementary material available at 10.1007/s00394-022-02935-7.

## Introduction

The effect of oral processing behaviour on bolus properties and its subsequent influence on nutrient digestion and utilization has been a growing research area during recent years [1, 2]. Especially in plant-based foods like cereals and legumes which are usually consumed as intact tissues, oral processing behaviour can modulate nutrient release from the cellular matrix. Inter-individual differences in oral behaviour can lead to different oral breakdown pathways of foods leading to differences in food bolus properties which may contribute to inter-individual differences in nutrient digestion and utilization [3–5].

Starch is one of the most essential macronutrients for humans. Many studies investigated the drivers of postprandial glucose responses and their relevance for metabolic disorders. Evidence shows that sustained higher blood glucose, and large spikes in postprandial glucose responses in a dietary pattern are risk indicators for type 2 diabetes and cardiovascular disease and are associated with obesity [6–8]. Postprandial glycaemic responses to the same food differ among individuals [9, 10]. To better understand these differences in postprandial glycaemic responses among individuals, the influence of oral processing behaviour on postprandial glycaemic responses to the same food has been investigated in several studies. In one study, young adults consumed a fixed amount of pizza with 15 or 40 chewing cycles. Chewing pizza 40 times resulted in higher concentrations of plasma glucose, insulin and glucose-dependent insulinotropic peptide (GIP) than chewing 15 times [11]. Ingesting small rice particles (500–1000 μm) produced greater glycaemic and insulin responses than ingesting large rice particles (> 2000 μm), suggesting that variability in oral processing behaviours between individuals contributes to differences in bolus properties which contribute to variability in glycaemic responses [12]. The glycaemic response to rice was inversely correlated with bolus particle size suggesting an important role of oral processing behaviour in moderating the degree of starch digestion of rice during the oral phase. However, this inverse association between bolus particle size and glycaemic response was not observed for spaghetti in the same study [5]. Another study demonstrated that longer chewing of white rice increased glycaemic responses suggesting that prolonged oral processing increased break down of the intact cell structure of rice. This increased saliva uptake in the bolus and accessibility of carbohydrates for digestive enzymes which may influence postprandial glucose responses [5, 13]. A recent study showed that eating fried rice slowly led to smaller bolus particle size, a larger bolus surface area, greater bolus saliva uptake and was associated with a higher postprandial glucose response [14].

The mechanisms underlying the impact of oral processing behaviours on postprandial glycaemic response are not fully understood. Food oral processing serves several functions. For solid foods, prolonged oral processing leads to changes in bolus properties including an increase in number of bolus particles, decrease in size of bolus particles, increase in total bolus surface area, and an increase in bolus saliva uptake [1, 2]. Increasing the specific bolus surface area can result in a higher accessibility of food bolus fragments to digestive enzymes [15]. Salivary amylase plays an additional role in the initial digestion of complex carbohydrates and their metabolism [16–18]. As outlined previously, different carbohydrate sources breakdown and metabolise at different rates, due to difference in their cellular structures. However, the combined contribution of oral processing behaviour, bolus properties and saliva to variations in postprandial glycaemic response to different carbohydrate sources remains unclear.

The current study sought to assess the impact of variations in oral processing behaviours (chewing time) of two starch-based foods that vary in fibre content and cell integrity (brown rice and chickpeas) on bolus properties, in vitro starch digestion and in vivo postprandial glycaemic response in normal healthy participants. In a second step we sought to verify the mediating roles of bolus properties including number, mean area of bolus particles and salivary amylase activity in in vitro starch digestion and in vivo postprandial glucose response. We hypothesize that longer chewing leads to formation of bolus with increased surface area and higher bolus saliva uptake which increases in vitro starch digestion and in vivo postprandial glycaemic response compared to shorter chewing.

## Materials and methods

### Participants and power calculation

Twenty-six participants were recruited from the database of Wageningen Food & Biobased Research for the in vivo study. A previous study by Madhu et al. [19] measured glucose responses before and after consumption of 25 g of groundnut using a normal and longer chewing protocol. Thorough mastication significantly reduced postprandial blood glucose levels 2 h after ingestion (128 ± 8 mg/dl for normal chewing vs 120 ± 9 mg/dl for longer chewing, *p* < 0.05). A power calculation was performed for the cross over design with an effect size of 8.5, a sigma of 9.1, alpha of 0.05 and power of 0.8 (http://hedwig.mgh.harvard.edu/sample_size/js/js_crossover_quant.html) which yielded 20 participants. Considering potential drop out, 26 participants, 13 per group, were enrolled in our study.

Participants (*n* = 26) were screened for suitability prior to consenting to join the study. Participants had the ability to chew and swallow normally, without history or undergoing treatment for chronic medical illness, smoking, alcohol or drug use, no type 1 or 2 diabetes, and no reported food allergies or intolerances to the tested foods. All participants (*n* = 26) were eligible, no participants were excluded and all participants (*n* = 26) completed the study. All participants gave written informed consent and received financial compensation for their participation in the study. The study was approved by the Medical Ethical Committee of Wageningen University (NL74340.081.20) and preregistered at clinicaltrials.gov (NCT04648397; ‘The effect of chewing duration on blood glucose levels’).

### Materials and preparation of test foods

Brown rice and chickpeas were chosen as test foods as they vary in fibre content and degree of cellular integrity at the moment of consumption. Test meals were matched for total carbohydrate content (Supplementary Table 1). Brown rice (precooked brown rice, Lassie BV, The Netherlands; hereafter referred to as brown rice (BR)), chickpeas (dried chickpeas, Jumbo Supermarkets BV, The Netherlands; hereafter referred to as chickpeas (CP)), spices (paprika and cinnamon; Euroma Baharat by Jonnie Boer, Koninklijke Euroma BV, The Netherlands) and rice oil (Alesie rice oil, AP Organics Ltd., India) were purchased from a local supermarket (Jumbo Supermarkets BV, The Netherlands) and stored at room temperature. All other chemicals used in this study were of analytical grade.

Test foods were cooked following the instructions provided on the product package. Brown rice was boiled for 8 min and allowed to stand covered with a lid for 5 min after draining the water. Chickpeas were boiled in water for 30 min after overnight soaking (12 h). Test lunches were prepared on each test day in the kitchen of the human research facility of Wageningen University and stored at 60 °C until consumption by participants. During the study, participants first received a portion of 9 g (one bite) brown rice or chickpeas for collection of bolus used for subsequent bolus analysis and then a bowl of 233 g brown rice or 323 g chickpeas (66 g carbohydrates per test meal) as test meals. Before serving, spices (1.25 ml per bowl) and rice oil (1 g per bowl) were added to the test meals to increase palatability.

### Characterization of chewing time for brown rice and chickpeas

Chewing time of brown rice and chickpeas was determined in a different group of *n* = 80 participants (45 females, 35 males, aged 28 ± 8 y). Gender, age, nationality, educational level, time living in the Netherlands, body weight and height, allergies and intolerances, dental status and smoking condition were collected from all participants using questionnaires. All participants (*n* = 80) were eligible, no participants were excluded and all participants (*n* = 80) completed this study. Chewing time was determined by providing a fixed bite size of 9 g of brown rice or chickpeas to participants. The bite size was established in a preliminary study as an average of the natural bite size when consuming test foods from a bowl (6 females, 8 males, aged 29 ± 6 y). Participants were instructed to chew naturally and swallow the test foods. The moment of swallowing was indicated by participants by raising their hand. Participants were video recorded during consumption. Chewing time (s) was extracted from the videos as the time period between placing the food into the mouth until swallowing. Number of chews was extracted from the videos and chewing frequency (chews/s) calculated. Participants were dived by median split into two groups corresponding to short and long chewing time. Table 1 shows the results of the median split. Chewing time of shorter and longer chewers was significantly different (*p* < 0.05) for both foods and differed by a factor of around 2 × between groups. Chewing time for short and long chewers was 23 and 41 s for brown rice, and 20 and 37 s for chickpeas. Chewing frequency (1.4 ± 0.2 chews/s) of brown rice and chickpeas was not significantly different between shorter and longer chewers and was not significantly different between foods (*p* > 0.05). The short and long chewing times shown in Table 1 were used in the main study to instruct participants for their chewing behaviour of the two test foods.

**Table 1:** Results of median split of chewing time (s) of fixed bite size (9 g) of brown rice and chickpeas

	Brown rice	Chickpeas
Average chewing time (s) *n* = 80	32 ± 13	28 ± 12
Short chewing time (s) *n* = 40	23 ± 4	20 ± 3
Long chewing time (s) *n* = 40	41 ± 13	37 ± 11

Chewing times of all participants (*n* = 80) and of short and long chewing time groups within each test food (*n* = 40 each group) are shown as mean ± SD

### Experimental design and food bolus collection

**Fig. 1:**
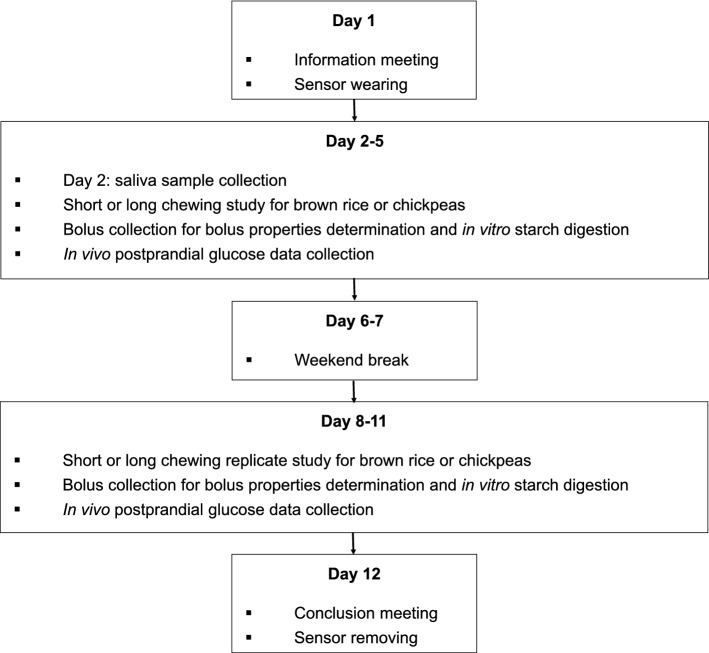
Schematical overview of study process

A semi-randomized cross-over trial (4 days per week in duplicate) was conducted and participants consumed two carbohydrates-identical test lunches of brown rice and chickpeas while either chewing for a short or long time. Test food consumption was randomized and balanced across all participants, and all participants completed all meals in a full cross-over design. Participants attended two formal test sessions within two weeks (Fig. 1). The first session was for training and initialization, which occurred on the Monday of the first study week (Day 1). During the first session, all study procedures were explained to all participants. At the end of this information session, a qualified research nurse placed a continuous glucose sensor (Freestyle Libre, CE certified, CE597686, Abbott BV, freestylelibre.com) on the participant’s arm. The starting time for the glucose recordings for every participant was always on the hour (10:00 am, 1:00 pm and 2:00 pm).

The second session was to provide and consume test lunches (Day 2–12). Before each test lunch, standard breakfast was handed to the participants one day before the test day. Participants consumed standard breakfasts before each test lunch before 8:00 am. The breakfast was composed by WUR dieticians and complied with the guidelines of the Voedingscentrum (National Dutch Nutrition center) in terms of energy content (300–400 kcals) and composition. The breakfast consisted of 2 slices of whole wheat bread, one cracker, 10 g of margarine, 15 g of jam, and 30 g of spreadable cheese and contained 348 kcals. Participants were allowed to drink a cup of water/tea/coffee (without milk and sugar) with their breakfast. At the beginning of each lunch session (Group 1: 12:00 pm; Group 2: 1:00 pm), research assistants downloaded the glucose data from the reader. Next, participants were asked to consume a fixed bite size of 9 g of each test food following chewing instructions provided in a video (see Sect. Characterization of chewing time for brown rice and chickpeas'). A 30 mL measuring cup filled with brown rice or chickpeas was used during the lunch session to fix bite to around 9 g per bite which is the weight corresponding to a 30 mL cup loosely filled with brown rice or based on the preliminary study (see Sect. Characterization of chewing time for brown rice and chickpeas'). The instruction video showed a person chewing the test food for 23 or 41 s for brown rice, or for 20 or 37 s for chickpeas with a constant chewing frequency of 1.4 chews/s (Table 1). The instruction video also provided a prompt tone every 0.7 s when a chew was taken. Participants watched the video with the prompt tones while masticating and were instructed to mimic the chewing behaviour shown in the video. The first bites were expectorated into containers at the moment of swallowing. Food boli were immediately stored on ice. Boli were subjected to subsequent bolus property measurements and in vitro digestion. Participants then consumed the full portion of the test lunch mimicking the oral behaviour that was shown in the instruction video. After finishing lunch, breakfast for the following session was handed to the participant. At 04:00 pm the participants were no longer restricted with regard to their food intake. On the last day of the study (Day 12), participants attended a concluding meeting to remove the sensor and to return the reader.

### Characterization of bolus properties

Particle number and particle size of bolus fragments were determined by image analysis [20]. In brief, 0.5 g of expectorated bolus was placed in a Petri dish (120 × 120 × 17 mm) and 25 mL of Milli-Q water was added to separate and distribute particles on the dish. All measurements were completed in duplicate. Individual bolus fragments were gently separated manually using a spatula. Petri dishes were placed on a flatbed scanner (Canon CanoScan 9000F MarkII) and a 600-dpi colour picture was taken with a black background. Pictures were imported into ImageJ (version 1.52a, National Institute of Health, USA) to conduct image analysis. Pictures were converted to an 8-bit image, after which a brightness/contrast adjustment and a black and white threshold were applied to obtain a binary image. For each image, the number of bolus particles (no./g) and bolus particle mean area (mm^2^/g) as a measure of bolus particle size were obtained and standardized per gram bolus. Particles smaller than 0.07 mm^2^ or with a circularity less than 0.15 were discarded from data analysis to prevent main interference of background [20].

### Determination of bolus saliva uptake

Dry matter content of brown rice and chickpeas and of their boli expectorated after short or long chewing time was determined in triplicate by drying a known amount of sample overnight in an oven at 105 °C to constant weight. Saliva uptake was calculated as follows [21].

$$Saliva\;{\mkern 1mu} uptake\left( \%  \right) = \left( {\frac{{m_{wet\;bolus}  - m_{dry\;bolus} }}{{m_{wet\;bolus} }} - \frac{{m_{wet\;test\;food}  - m_{dry\;test\;food} }}{{m_{wet\;test\;food} }}} \right) \times 100\%$$

### Calculation of salivary amylase activity in food bolus

Expectorated stimulated saliva was collected on the first day from all participants after they chewed on a piece of parafilm (3 × 3 cm, Parafilm M PM996) for 5 min. Samples were stored at − 80 °C before they were analysed [16]. Salivary amylase activity (U/mL) in stimulated saliva was determined by α-amylase saliva colorimetric assays (RE80111, IBL International GmbH, Hamburg, Germany). The intensity of the colour developed was proportional to the activity of α-amylase in the sample. Absorbance at 405 nm was measured at 37 °C using a spectrophotometer. Next, saliva amylase activity in food bolus (U/g) was estimated by the following equation [16] in which saliva density was taken as 1.00 g/mL [18].

$$Salivary\;{\mkern 1mu} amylase{\mkern 1mu} \;activity\;{\mkern 1mu} in\;food\;{\mkern 1mu} bolus{\mkern 1mu} \left( {U/g} \right){\mkern 1mu}  = {\mkern 1mu} \frac{{Saliva\;{\mkern 1mu} uptake{\mkern 1mu} \left( \%  \right)}}{{Saliva\;{\mkern 1mu} density{\mkern 1mu} \left( {g/mL} \right)}}\quad \quad  \times \alpha lpha\;amylase\;activity(U/mL)$$

### In vitro starch digestion

Expectorated brown rice and chickpeas boli after short and long chewing time were collected from a variable number of participants (8–16 participants, depending on the food and the chewing duration, varying due to unpredictable absence and sample contamination). Boli were subjected to in vitro starch digestion immediately after expectoration. In vitro digestion was performed for each bolus according to the harmonized INFOGEST 2.0 protocol [22]. Pepsin (P7012, 2,500–3,000 units/mg protein), pancreatin (P1750, 4X USP specification) and porcine bile extract (B8631) were purchased from Sigma-Aldrich Ltd (St Louis, MO, USA). Enzymes used in simulated digestive fluids were assayed for their activity and diluted to reach the required activity based on the INFOGEST 2.0 protocol [22]. First, the food bolus was weighed and diluted 1:1 w/w with Milli-Q water to reach the same volume as the in vitro oral digesta described in INFOGEST 2.0, e.g. 5.1 g bolus + 5.1 mL Milli-Q water. Secondly, the mixture was added to prewarmed simulated gastric fluid (SGF) and pepsin (2000 U/mL final concentration). The pH was adjusted to 3 with 5 M HCl before 2 h incubation at 37 °C to simulate the gastric phase. In vitro digestion was performed in a laboratory incubator with constant rotation by rotator. Aliquots from the supernatant (0.1 mL) were taken after 0 and 120 min, and 0.4 mL of ethanol was added and shaken in a vortex mixer for 10 s to stop gastric digestion.

For intestinal digestion, gastric chyme (the whole content of the tube after gastric digestion) was combined with prewarmed simulated intestinal fluid (SIF), pancreatin (200 U/mL for amylase in pancreatin final concentration) and bile salts (10 mM final concentration). The pH was raised to 7 with 5 M NaOH before incubation at 37 °C for another 2 h. In vitro digestion was performed in a laboratory incubator under constant rotation by rotator. Three aliquots from the supernatant were taken during the intestinal digestion (15, 30 and 120 min). The values for the end points of gastric digestion at 120 min correspond to the values of the intestinal digestion at 0 min. To stop intestinal digestion, 0.4 mL of ethanol was added to each intestinal aliquot and shaken in a vortex mixer for 10 s. All digestion aliquots were allowed to rest for 30 min and stored into -20 °C freezer until further analysis.

For the determination of the degree of in vitro starch hydrolysis, the ethanolic solution aliquots were thawed and centrifuged at 10,000 ×*g* for 10 min. Then 0.1 mL of ethanolic supernatant was incubated with 0.5 mL amyloglucosidase solution (27 U/mL) in acetate buffer (0.1 M, pH 4.8) for conversion of α-amylase products into glucose at 37 °C for 1 h [23, 24]. D-glucose assay procedure (GOPOD-FORMAT, K-GLUC 04/20, Megazyme Ltd., Bray, Wicklow, Ireland) was used to quantify the amount of glucose present at each digestive time point. Glucose content was converted into the corresponding amount of starch by multiplying by a factor of 0.9. Total starch content in brown rice and chickpeas was determined by the Total Starch (AA/AMG) test kit from Megazyme Ltd (Bray, Wicklow, Ireland). Results of in vitro degree of starch hydrolysis (DH_Starch%) were presented as percentage of hydrolyzed starch in total dry starch of test food matrix and food bolus.

### In vivo postprandial glycaemic response

In vivo postprandial glycaemic responses were monitored for all participants (*n* = 26) using continuous blood glucose monitors (Freestyle Libre, CE certified, CE597686, Abbott BV, freestylelibre.com) during the 12-day study period. On 8 of 12 days, participants consumed the controlled test meals in duplicate. The Freestyle Libre system consists of two parts, the first is the sensor and the second is the reader. The sensor was placed on participant’s arms one day before the start of the test period to allow the sensor to stabilize (Day 1, see Sect. 'Experimental design and food bolus collection'). Participants received the instructions on how to use the reader during the first session of the study. Participants received a reader which was used every day at 04:00 pm to transfer the glucose measurements from the monitor to the reader. Furthermore, they were instructed to do a scan upon arrival of the test day, before the test lunch.

Glucose levels were automatically recorded continuously with the sensor every 15 min for the whole intervention period, and were transferred to the reader every 8 h. For this study, only the data between breakfast and the 4-h post-lunch interval was used. Participants were instructed via a phone message to download and transfer the data from the sensor to the reader at 04:00 pm. The direct output of the in vivo study was postprandial glucose concentration. The primary outcome was incremental area under the curve (iAUC) for blood glucose. The iAUC for glucose after test lunch was calculated by the trapezoidal method.

Peak postprandial blood glucose (PPG) concentration, mean time to postprandial peak blood glucose concentration and incremental area under the curve (iAUC) for early PPG 0–30 min, and later PPG 30–150 min and four-hour postprandial blood glucose were extracted and calculated from the dataset. The original dataset was downloaded from the continuous glucose sensor in txt and pdf files. To extract the data, Microsoft Excel was used to transform and perform the calculations. These characteristics of postprandial glycaemic parameters were analysed for participants after consuming the two test lunches of brown rice and chickpeas with either short or long chewing time (Table 1).

### Statistical data analysis

To investigate the influence of chewing time and food type on bolus properties, linear mixed models were used with food type (brown rice and chickpeas) and chewing time as fixed factors and participant as random effect.

To investigate the influence of chewing time and food type on in vitro degree of starch hydrolysis linear mixed models were used with food type, chewing time, digestion time, and gender as fixed effects and participant as random effect.

To investigate the influence of chewing time and food type on in vivo glycaemic response (iAUC), linear mixed models were used with food type, chewing time, and digestion time as fixed effects and participant as random effect.

Bivariate Pearson correlation tests (two-tailed) were used to examine the relationships between salivary amylase activity or bolus properties (number and mean area per gram of bolus) and in vitro degree of starch hydrolysis or in vivo glycaemic response as well as the relationships between in vitro degree of starch hydrolysis at *t* = 0 and in vivo glycaemic response for each of the two test foods consumed with short or long chewing time.

Comparisons between characteristics of postprandial glycaemic parameters of brown rice and chickpeas consumed with short and long chewing time, including peak postprandial blood glucose concentration, mean time to postprandial peak blood glucose concentration and iAUC for postprandial blood glucose, were performed assuming heterogeneity of variances with post hoc Games-Howell multiple comparison tests.

The results are expressed as mean ± standard deviation (SD) unless otherwise stated. A significance level of *p* < 0.05 was chosen. IBM SPSS Statistics (version 25.0) was used to perform all statistical analysis.

## Results

### Influence of chewing time on bolus properties of brown rice and chickpeas

**Fig. 2:**
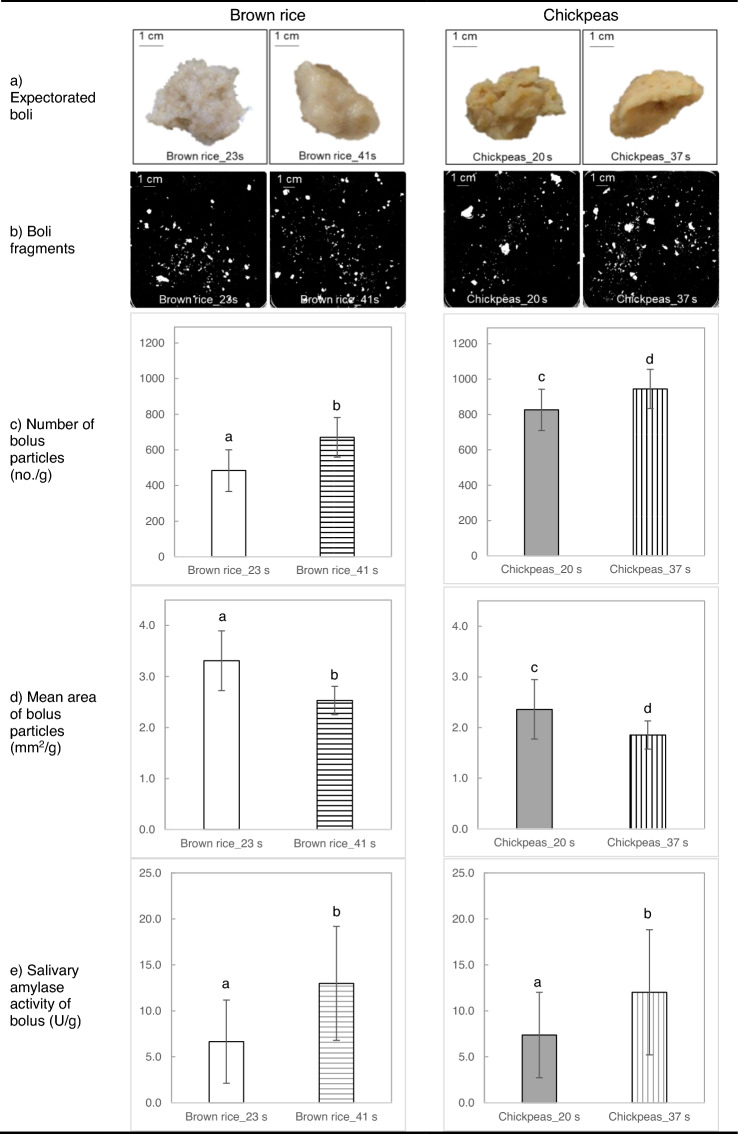
Bolus properties of brown rice and chickpeas chewed for short and long time. Brown rice chewed for 23 or 41 s; Chickpeas chewed for 20 or 37 s. **a** Representative pictures of expectorated boli of one participant. **b** Representative scans of separated boli particles of one participant. **c** Number of bolus particles (mean ± SD; Brown rice_23 s: *n* = 12; Brown rice_41 s: *n* = 16; Chickpeas_20 s: *n* = 8; Chickpeas_37 s: *n* = 16; duplicate). **d** Mean area of bolus particles (mean ± SD; Brown rice_23 s: *n* = 12; Brown rice_41 s: *n* = 16; Chickpeas_20 s: *n* = 8; Chickpeas_37 s: *n* = 16; duplicate). **e** Salivary amylase activity of bolus (U/g) (mean ± SD; Brown rice_23 s: *n* = 16; Brown rice _41 s: *n* = 13; Chickpeas_20 s: *n* = 13; Chickpeas_37 s: *n* = 13) Number (no.) and mean area (mm^2^) of bolus particles were normalized by weight (g) of scanned bolus. Different letters indicate significant differences between means between short and long chewing time (*p* < 0.05)

In Fig. 2 representative images of expectorated boli and scans of separated boli particles of brown rice and chickpeas as well as bolus properties (number and mean area of bolus particles) are shown. For both test foods, longer chewing resulted in significantly more (*F*(2, 39.6) = 4.6, *p* = 0.016) and smaller (*F*(2, 39.3) = 20.4, *p* < 0.001) bolus fragments than shorter chewing. Brown rice displayed significantly less (*F*(1, 36.3) = 46.7, *p* < 0.001) and larger (*F*(1, 35.2) = 81.6, *p* < 0.001) bolus particles than chickpeas regardless of chewing time. In addition, the boli chewed for longer time showed significantly higher salivary amylase activity (*F*(2, 51) = 6.9, *p* < 0.01) than boli chewed for shorter time regardless of food type.

### Influence of chewing time, salivary amylase activity and bolus properties on in vitro starch hydrolysis of brown rice and chickpeas

**Fig. 3:**
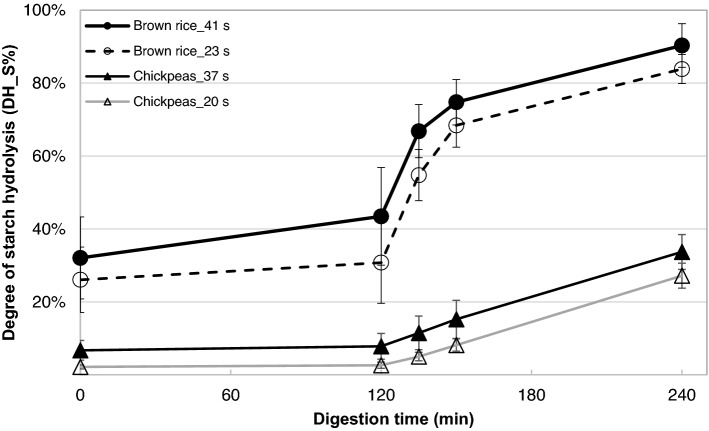
In vitro degree of starch hydrolysis (DH_S%) of brown rice and chickpeas. Brown rice chewed for 23 or 41 s; Chickpeas chewed for 20 or 37 s. Brown rice_41 s is reported as mean ± SD of *n* = 16 participants. Brown rice_23 s is reported as mean ± SD of *n* = 12 participants. Chickpeas_37 s is reported as mean ± SD of *n* = 8 participants. Chickpeas_20 s is reported as mean ± SD of *n* = 16 participants

Figure 3 shows the in vitro degree of starch hydrolysis (DH_S%) of brown rice and chickpeas chewed for short and long time.

Chewing time had a significant effect on in vitro starch digestion (*F*(2, 183.8) = 8.7, *p* < 0.001). In vitro degree of starch hydrolysis (DH_S%) (*t* = 240 min) at the end of the simulated intestinal digestion of brown rice chewed long (41 s; DH_S% = 90.3%) was 6.4% higher (in absolute terms) than of brown rice chewed shortly (23 s; DH_S% = 83.9%). Similarly, DH_S% (*t* = 240 min) at the end of the simulated intestinal digestion of chickpeas chewed long (37 s; DH_S% = 33.7%) was 6.5% higher than of chickpeas chewed shortly (20 s; DH_S% = 27.2%). For both test foods, longer chewing time resulted in significantly higher DH_S% than shorter chewing time. Brown rice had a significantly higher degree of starch hydrolysis than chickpeas (*F*(1, 190.5) = 1465.7, *p* < 0.001) regardless of chewing time. DH_S% of brown rice chewed short (23 s) was 56.7% higher than for chickpeas chewed short (20 s) at the end of in vitro starch digestion (t = 240 min). Gender (*F*(1, 15.4) = 2.6, *p* = 0.13) did not significantly affect DH_S%.

Table 2 shows correlations between salivary amylase activity of bolus (U/g) and in vitro degree of starch hydrolysis (DH_S%) of brown rice and chickpeas chewed short and long at the beginning of the simulated gastric phase (0 min), the end of the simulated gastric phase (120 min) and the end of the simulated intestinal phase (240 min). Salivary amylase activity was significantly and positively correlated (*p* < 0.01) with DH_S% of brown rice at the beginning (*t* = 0 min) and end point (*t* = 120 min) of in vitro starch gastric digestion, regardless of chewing time. No additional significant correlations (*p* > 0.05) were found between salivary amylase activity and DH_S% of neither test food (brown rice, chickpeas) for any chewing condition (short, long) at any digestion time point. No correlations were observed between bolus properties (number and mean area of bolus particles per gram bolus) and DH_S% of brown rice and chickpeas for any chewing time (short, long).

**Table 2:** Correlations between salivary amylase activity (U/g) and in vitro degree of starch hydrolysis (DH_S%) of brown rice and chickpeas chewed for short and long time

Sample		Correlation between saliva amylase activity of bolus (U/g) and in vitro degree of starch hydrolysis (DH_S%)		
	Digestion time/min	*r*		
Brown rice_23 s	0	0.874		**
	120	0.898		**
	240	0.391		NS
Brown rice_41 s	0	0.727		**
	120	0.689		**
	240	0.296		NS
Chickpeas_20 s	0	0.464		NS
	120	0.404		NS
	240	− 0.249		NS
Chickpeas_37 s	0	0.244		NS
	120	0.302		NS
	240	0.455		NS

Brown rice chewed for 23 or 41 s; Chickpeas chewed for 20 or 37 s. Digestion time 0 min: Beginning of simulated gastric phase; 120 min: End of the simulated gastric phase; 240 min: End of simulated intestinal phase (240 min). Derived by bivariate Pearson correlation (two-tailed)

Significance level is presented as NS (non-significant); *(*p* < 0.05), **(*p* < 0.01), and ***(*p* < 0.001)

### Influence of chewing time, salivary amylase activity and bolus properties on in vivo postprandial glycaemic response of brown rice and chickpeas

**Fig. 4:**
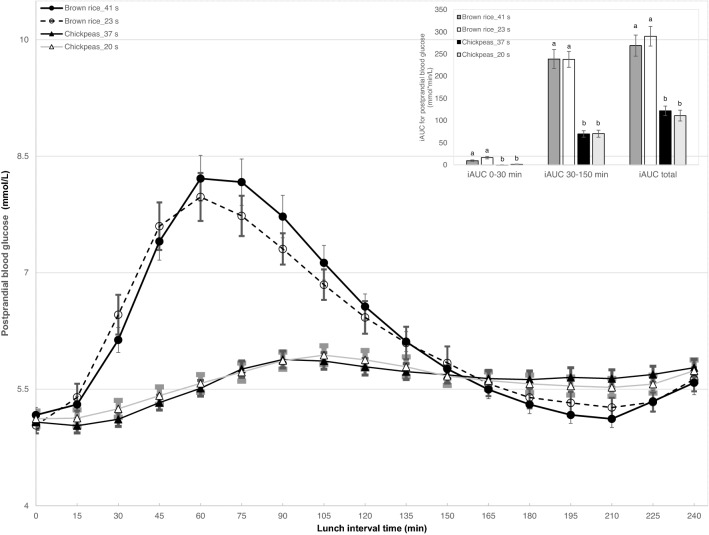
Postprandial glycaemic response for four hours after lunch of brown rice and chickpeas chewed for short and long time. Brown rice chewed for 23 or 41 s; Chickpeas chewed for 20 or 37 s. Insert shows the incremental area under the curve (iAUC) for postprandial blood glucose at 0–30 min, between 30 and 150 min and total iAUC (0–240 min). Data is reported as mean ± SEM of *n* = 26 participants. Letters (**a**, **b**) indicate significant difference at *p* < 0.05 (Games-Howell multiple comparison tests)

Figure 4 shows the four-hour postprandial glycaemic response after consumption of a test lunch of brown rice and chickpeas chewed for short and long time, respectively.

Chewing time had no significant effect on the glycaemic excursion after consumption of brown rice or chickpeas (*p* > 0.05). The bar chart insert indicated no significant difference in iAUC for blood glucose of brown rice or chickpeas in the early (0–30 min), later (30–150 min) and total (0–240 min) postprandial period between longer and shorter chewing times (*p* > 0.05). As expected, brown rice showed a significantly higher postprandial blood glucose response than chickpeas for both chewing times (*p* < 0.001). Peak postprandial blood glucose concentration and mean time to postprandial peak blood glucose concentration of brown rice were significantly higher than those for chickpeas (Table 3, *p* < 0.01). In contrast to the in vitro result, chewing time had no significant influence on these glycaemic parameters (*p* > 0.05).

**Table 3:** Postprandial glycaemic parameters extracted from blood glucose concentration profiles after consumption of brown rice and chickpeas with short and long chewing time. Brown rice chewed for 23 or 41 s; Chickpeas chewed for 20 or 37 s. Data is reported as mean ± SD of *n* = 26 participants

Glycaemic parameters	Brown rice_23 s	Brown rice_41 s	Chickpeas_20 s	Chickpeas_37 s
Peak postprandial blood glucose concentration (mmol/L)	8.40 ± 1.68^a^	8.65 ± 1.73^a^	6.23 ± 0.78^b^	6.21 ± 0.72^b^
Mean time to postprandial peak blood glucose concentration (min)	72 ± 31^a^	83 ± 43^a^	146 ± 65^b^	149 ± 69^b^

*iAUC* Incremental area under the curve

The superscripted letters, ^a^ & ^b^, indicate significant differences (*p* < 0.01, Games-Howell multiple comparison tests)

No significant correlation was observed between saliva amylase activity and in vivo glucose response for the two test foods between longer and shorter chewing times. No significant correlation (*p* > 0.05) was found between bolus properties (number and mean area per gram bolus) and in vivo glucose response of brown rice or chickpeas under any longer or shorter chewing time.

## Discussion

In this study, we investigated the impact of oral processing time on bolus properties, in vitro starch digestion and postprandial glycaemic response of two carbohydrate foods that differ in cellular structure. The results demonstrate that for brown rice and chickpeas, differences in chewing time led to differences in bolus surface area, saliva uptake and in vitro starch digestion, but chewing time had no effect on in vivo postprandial blood glucose responses. Differences in the innate cellular structure and composition between brown rice and chickpeas had a larger effect on postprandial glucose responses compared to differences in mastication behaviour.

A significant effect of chewing time on in vitro starch digestion was found, though this result was not observed in vivo. This finding supported our initial hypothesis that a longer chewing time increases the in vitro degree of starch hydrolysis but rejected our hypothesis that PPG increases. Prolonged chewing resulted in bolus breakdown into smaller bolus fragments with a larger surface area (Fig. 2), which likely increased accessibility of amylase to starch within bolus particles [15]. In addition, the longer chewing time increased saliva uptake and the available time for saliva penetration of the food bolus, supporting a higher degree of starch hydrolysis. Figure 3 showed that a substantial amount of starch is already degraded at the beginning of simulated gastric digestion (*t* = 0 min, DH_S%_23 s_ = 26% and DH_S%_41 s_ = 32%). This starch hydrolysis may be caused by salivary amylase hydrolysing starch during handling of the samples before performing the in vitro digestion experiments. Previously it was shown that salivary amylase is able to hydrolyse up to 80% of wheat bread starch during the first 30 min of in vitro gastric digestion [25]. The current study did not find a significant relationship between salivary amylase activity and in vitro DH_S% (Table 2). The studies that find a significant contribution of salivary amylase tend to be studies that compare participants with extremely low and high salivary amylase activity [16], while studies that determined these correlations in a conveniently large sample from the population failed to show these relationships [17, 26]. One possible reason for the higher degree of DH_S% at *t *= 0 min in brown rice compared to chickpeas may be that the cells were more extensively damaged in brown rice than chickpeas. Boiling is known to produce cell breakage in cooked rice because of starch expansion during gelatinization, while cell walls are more resistant to cooking in chickpeas [27, 28]. In addition, the relationships between DH_S% (*t* = 0) and PPG (iAUC: 0–30 min, 30–150 min and total/240 min) were also analysed (Supplementary Table 2). All correlations between DH_S% (*t* = 0) and PPG at the different time points were not significant except for brown rice chewed for short time, for which DH_S% (*t* = 0) correlated significantly with iAUC 30–150 min (*p* = 0.05, *r* = 0.667). The current results therefore did not provide strong evidence for an alignment between DH_S% (*t* = 0) with PPG. Overall, the difference in DH_S% caused by different chewing time was maintained until the end of in vitro intestinal digestion.

The effect of chewing time on in vitro starch digestion was not observed in vivo, where chewing time did not affect postprandial glycaemic responses. Several studies reported no significant effect of chewing time on plasma glucose response [29–31]. One possible explanation for the lack of an effect of chewing time on postprandial glycaemic response is the homeostatic regulation of physiological functions in generally healthy young participants. Healthy, young individuals should have an efficient glucose regulatory system, which can modulate postprandial hormone response to avoid considerable glycaemic fluctuation by the difference in chewing time [32]. Another possible reason may be the lack of sensitivity of the in vivo continuous glucose monitor to subtle changes in PPG, which is more designed for tracking changes in blood glucose over an extended period of time, rather than providing an absolute measure of blood glucose concentration at a given time-point. There are diverging results concerning the effect of chewing time on in vivo postprandial responses including glycaemic response and hormone response. Several studies showed that longer chewing leads to higher glycaemic responses and/or insulinemic response [11, 13, 33]. One study reported that among participants with a higher risk for type 2 diabetes, increasing oral processing time of fried rice led to a greater bolus fragment surface area, more saliva uptake in bolus and higher postprandial glucose and insulin responses [14]. Whereas another study reported that chewing white rice for 10 times per bite compared to 40 times resulted in no effect on glycaemic response but lower insulinemic response [34]. One study demonstrated that longer chewing elicited a significantly lower postprandial plasma glucose concentration as well as a higher insulin response in healthy participants after consuming hamburgers and rice [4]. These results suggest that glucose metabolism is a multifactorial and dynamic process in which chewing time is only one contributing factor. The difference between in vivo and in vitro results in the current trial suggests the extent to which chewing time can influence glucose metabolism is likely to be subtle, and is a product of the simultaneous action of multiple factors including health status of participants, food composition, intrinsic cellular food structure, processing status (e.g., milling) and cooking status of the test food [35]. The discrepancy between in vitro starch digestion and in vivo postprandial glucose responses suggests that modifications in the in vitro model of digestion might be necessary to produce results that are predictive for postprandial responses.

The present study showed no correlation between bolus properties and in vitro degree of starch hydrolysis or in vivo postprandial glycaemic response, which is in line with our previous work on proteins [20]. We hypothesized there would be an inverse correlation between particle size and degree of starch digestion among plant foods like cereals and legumes, because a more intense chewing reduces particle size, increases fracture of intact cells and therefore increases digestive enzymes accessibility to substrates [5, 20, 35–40]. In addition, we also investigated the potential contribution of salivary amylase activity and showed that within group variation in salivary amylase activity was not significantly related with in vitro starch digestion or in vivo postprandial glycaemic response for neither chewing group. This suggests that salivary amylase activity may influence glycaemic response, but is likely to be most influential during the early phase of digestion. This is in agreement with amylolysis of wheat bread starch during in vitro gastric digestion reported elsewhere [25]. The lack of correlation between salivary amylase activity and in vitro starch digestion or in vivo postprandial glycaemic response suggests that the variability in salivary amylase activity observed in our study had a negligible effect. Within a healthy population, the differences in chewing times and bolus properties on in vivo postprandial glycaemic response may be counteracted by an efficient glucose homeostatic regulation system. An advantage of studying healthy participants is that it allows to directly compare chewing regimes without the additional confounding effect of an underlying clinical condition. However, differences in oral processing are likely to be more influential among specific populations such as pre-clinical diabetic populations or older consumers where further research is needed to understand the role of chewing and bolus properties on nutrient digestion.

The test meal had a significant influence on bolus properties, in vitro degree of starch hydrolysis and in vivo postprandial glycaemic response, where longer chewing of brown rice produced smaller bolus fragments and a larger bolus surface area. Comparing the two test foods, brown rice resulted in less and larger bolus particles than chickpeas, even after the longer chewing time (23 s vs 20 s and 41 s vs 37 s). These differences in bolus properties were related to the texture of the test foods. Hard and dry solid foods need longer chewing time than softer solid foods [41–43] and the hard, dry texture of brown rice required longer chewing time which generated larger bolus particles than chickpeas. There was a substantially higher degree of in vitro starch digestion and a higher postprandial glucose response for brown rice than chickpeas, regardless of chewing time, despite the fact that both test lunches were fixed for the total amount of carbohydrates. It is also noteworthy that chewing time had an impact on the temporal changes in glucose for brown rice compared to chickpeas (Fig. 4 and Table 3), albeit not significant, there is a stronger effect in brown rice and in line with previous findings so longer chewing may result in different degree of impact between test meals [5]. The two test foods varied in fibre content with 3 g/portion for cooked brown rice and 15 g/portion for cooked chickpeas. In addition to fibre content, differences in cell integrity between the two test foods that may also produce differences in starch digestibility [28]. Dietary fibre occurs in the cell walls of plant cells and fibre cannot be digested in humans. For this reason, a higher degree of cell integrity might reduce postprandial glycaemic responses by restricting digestive enzyme accessibility to starch granules [44, 45]. In the current study, cell walls are likely to be more extensively broken down in brown rice during boiling compared to chickpeas [46].The two test foods also differed in amylose content (brown rice contains around 20% of amylose starch and chickpeas contains around 30%) [47–49]. In general, starch digestion is reported to be slower when the amylose content of starch increases [50]. The glycaemic response to starch also depends on the degree of starch gelatinization [50]. Moist heat cooking gelatinizes starch and increases starch digestibility and the resultant glycaemic response [51]. In this regard, the brown rice test meal may contain more gelatinized starch compared to chickpeas where the relatively rigid and resistant cell wall prevents full swelling of starch granules thus reducing its digestibility [27, 28]. A better understanding of how differences in cell structure and cooking preparation method interact with oral processing behaviours, will provide new insights and guidance for how to moderate post-ingestive glycaemic flux in the future.

In conclusion, we demonstrated that structural and composition differences between the test foods (brown rice and chickpeas) had a stronger impact on postprandial glucose release and in vitro starch digestion than chewing time despite prolonged chewing increasing surface area and saliva uptake of bolus. The discrepancy between in vitro and in vivo starch digestion results of brown rice and chickpeas shows the importance of comparison and validation of in vitro methods with in vivo studies which requires more and larger investigations in the future.

The current study highlights that oral processing behaviour can have a consistent effect on in vitro macronutrient digestion though differences in the innate structure of the starch based foods has a larger effect on postprandial glucose release. Further research is needed to better understand the relationship between oral processing behaviour and macronutrient digestion and to clarify the potential for oral processing behaviour to be applied as an intervention target to enhance postprandial metabolic responses to specific nutrients. For instance, whether differences in oral processing behaviour produced by incorporating test food into complex meal may contribute to differences in nutrient digestion, or whether differences in oral processing behaviour of specific populations, such as older consumers or pre-diabetics, lead to differences in digestion.

## Supplementary Information

Below is the link to the electronic supplementary material.

Supplementary file1 (PDF 27 KB)

## Data Availability

The datasets used and/or analysed during the current study are available from the corresponding author on reasonable request.
