# Supplementary material for: Influence of oral processing behaviour and bolus properties of brown rice and chickpeas on in vitro starch digestion and postprandial glycaemic response
Source: Eur J Nutr. 2022 Jun 30;61(8):3961–74. doi: 10.1007/s00394-022-02935-7 (PMC9596526; doi:10.1007/s00394-022-02935-7)
Supplement: Supplementary file 1 — Supplementary file1 (PDF 27 KB) [file 394_2022_2935_MOESM1_ESM.pdf]

Supplementary table 1 Nutritional information of test lunches of brown rice and chickpeas (Nutrients information is available from: https://nevo-online.rivm.nl/. Glycemic load is available from: https://www.health.harvard.edu/ & https://www.voedingscentrum.nl/nl.aspx).

Test lunch	Portion size/g	Calories/kCal	Fat/g	Carbohydrates/g	Polysaccharides/g	Fibre/g	Glycaemic load
Brown rice (cooked)	232	315	2	66	62	3	45
Rice oil	1	9	1	0	0	0	
Total	233	324	3	66	62	3	
Chickpeas (cooked)	323	445	3	66	60	15	19

Supplementary table 2 Correlations between *in vitro* degree of starch hydrolysis (DH_S%) at digestion time=0 min (Beginning of simulated gastric phase) of brown rice and chickpeas chewed for short and long time and the incremental area under the curve (iAUC) for postprandial blood glucose at 0-30 min, between 30-150 min and total iAUC (0-240 min). Brown rice chewed for 23 or 41 s; Chickpeas chewed for 20 or 37 s. Derived by bivariate Pearson correlation (two-tailed).

Sample		Correlation between *in vitro* degree of starch hydrolysis (DH_S%, t=0) and iAUC	
DH_S%, t=0	iAUC	*r*	
	0-30	0.320	NS
Brown rice_23 s	30-150	0.667	*
	total	0.526	NS
	0-30	0.193	NS
Brown rice_41 s	30-150	0.127	NS
	total	0.145	NS
	0-30	-0.508	NS
Chickpeas_20 s	30-150	0.014	NS
	total	0.135	NS
	0-30	-0.304	NS
Chickpeas_37 s	30-150	-0.077	NS
	total	-0.024	NS

Significance level is presented as NS (non-significant); *($p < 0.05$), **($p < 0.01$), and ***($p < 0.001$).
